# Complete Genome Sequence of *Francisella tularensis* Subspecies *holarctica* FTNF002-00

**DOI:** 10.1371/journal.pone.0007041

**Published:** 2009-09-16

**Authors:** Ravi D. Barabote, Gary Xie, Thomas S. Brettin, Steven H. Hinrichs, Paul D. Fey, Justin J. Jay, Jennifer L. Engle, Shubhada D. Godbole, Jyothi M. Noronha, Richard H. Scheuermann, Liwei W. Zhou, Christine Lion, Michael P. Dempsey

**Affiliations:** 1 Bioscience Division, M888, Los Alamos National Laboratory, Los Alamos, New Mexico, United States of America; 2 DOE Joint Genome Institute (JGI), Walnut Creek, California, United States of America; 3 Department of Pathology and Microbiology, University Nebraska Medical Center, Omaha, Nebraska, United States of America; 4 Division of Microbiology, Armed Forces Institute of Pathology, Washington, D. C., United States of America; 5 BioHealthBase/Department of Pathology, UT Southwestern Medical Center, Dallas, Texas, United States of America; 6 BioHealthBase/Northrop Grumman Information Technology, Rockville, Maryland, United States of America; 7 Laboratoire de Bactériologie, Centre Hospitalier et Universitaire, Nancy, France; University of Hyderabad, India

## Abstract

*Francisella tularensis* subspecies *holarctica* FTNF002-00 strain was originally obtained from the first known clinical case of bacteremic *F. tularensis* pneumonia in Southern Europe isolated from an immunocompetent individual. The FTNF002-00 complete genome contains the RD_23_ deletion and represents a type strain for a clonal population from the first epidemic tularemia outbreak in Spain between 1997–1998. Here, we present the complete sequence analysis of the FTNF002-00 genome. The complete genome sequence of FTNF002-00 revealed several large as well as small genomic differences with respect to two other published complete genome sequences of *F. tularensis* subsp. *holarctica* strains, LVS and OSU18. The FTNF002-00 genome shares >99.9% sequence similarity with LVS and OSU18, and is also ∼5 MB smaller by comparison. The overall organization of the FTNF002-00 genome is remarkably identical to those of LVS and OSU18, except for a single 3.9 kb inversion in FTNF002-00. Twelve regions of difference ranging from 0.1–1.5 kb and forty-two small insertions and deletions were identified in a comparative analysis of FTNF002-00, LVS, and OSU18 genomes. Two small deletions appear to inactivate two genes in FTNF002-00 causing them to become pseudogenes; the intact genes encode a protein of unknown function and a drug:H^+^ antiporter. In addition, we identified ninety-nine proteins in FTNF002-00 containing amino acid mutations compared to LVS and OSU18. Several non-conserved amino acid replacements were identified, one of which occurs in the virulence-associated intracellular growth locus subunit D protein. Many of these changes in FTNF002-00 are likely the consequence of direct selection that increases the fitness of this subsp. *holarctica* clone within its endemic population. Our complete genome sequence analyses lay the foundation for experimental testing of these possibilities.

## Introduction

*F. tularensis*, the etiologic agent of tularemia, is a Gram-negative, facultative intracellular zoonotic pathogen originally isolated from ground squirrels in 1911 during a plague investigation in Tulare County, CA [1]. It is known to infect numerous animal species including humans [2], [3]. The organism has been weaponized and is currently listed as a Category A Select Agent posing a significant threat to humans especially due to its extremely low infectious dose when acquired through the inhalation route [4].

*F. tularensis* is comprised of three subspecies: subsp. *tularensis* (also Type A or *F.t. tularensis* hereafter), subsp. *holarctica* (also Type B or *F.t. holarctica*), and subsp. *mediaasiatica*. *F. novicida*, officially considered a separate species, is often considered a fourth *F. tularensis* subspecies and will be treated as such in this publication [5]. Epidemiology suggests only the *tularensis* and *holarctica* subspecies are clinically significant in humans [6], and between them it is believed that Type A causes more serious disease [7], [8].

Comparative genome hybridization (CGH) studies have demonstrated small differences between *F.t. tularensi*s and *F.t. holarctica* populations based on genome contents. Eight regions of difference (RD) were found to distinguish all *F.t. tularensis* from *F.t. holarctica* strains tested from a geographically diverse collection [9], [10]. These RD include the subsp. *tularensis*-specific RD*_tularensis_*6, a segment of the *Francisella* Pathogenicity Island (FPI) encompassing the *pdpD* gene shown to be involved in virulence differences between *F. tularensis* subspecies [11], and which is truncated and non-functional in *F.t. holarctica*. In addition, other comparative genome studies have shown extensive genome rearrangements including translocations and inversions between *F.t. tularensis* and *F.t. holarctica* [12], [13]. These changes were highly conserved in strains from multiple geographic locations, particularly in *F.t. holarctica* populations, indicating a likely early origin during subspecies divergence [12]. The rearrangement events likely caused changes in expression patterns of genes adjacent to the rearrangement sites, possibly explaining some of the virulence differences between the two subspecies.

Beyond differences in virulence, the *F. tularensis* subspecies also differ in geographic distribution, with *F.t. tularensis* largely limited to North America whereas *F.t. holarctica* has been documented throughout the N. Hemisphere [14]. Within the *tularensis* subspecies, genetic diversity is well documented, with distinct genetic groups A.I and A.II found in central and western parts of the U.S., respectively [3], [8], [15]. Genetic analyses of *F.t. holarctica* strains from globally derived populations showed that strains from different continents could similarly be found in nearly all of the major *F.t. holarctica* clusters [15]. Collectively, these studies suggest *F.t. holarctica* may be relatively newer and genetically more homogeneous than *F.t. tularensis*.

With respect to *F.t. holarctica*, phylogeographic genome variation has been detected among global *F.t. holarctica* populations [16], [17] including parts of Europe as demonstrated by a new RD, termed RD_23_. RD_23_ was found in all DNA samples from a subset of strains from Spain and France, which by phylogenetic analysis comprise a highly clonal population likely to have recently emerged on the Iberian Peninsula [16]. Most of the strains analyzed were from an epidemic outbreak primarily in rabbits and humans in Spain between 1997–1998, and which was the first reported tularemia outbreak in that country [14], [18]. The outbreak involved over 500 human cases of tularemia [19], [20], [21], [22]; as expected given the geographic location, all samples tested were identified as *F.t. holarctica* [18], [21], [23], [24].

In the present work, we report on the first complete genome sequence (CGS) of *F.t. holarctica* strains carrying RD_23_. The sequence, designated FTNF002-00, is from a strain isolated from a previously published clinical case in France involving an immunocompetent 56-year old male with bacteremic *F.t. holarctica* pneumonia [25]. FTNF002-00 revealed several genomic differences with respect to other published *F.t. holarctica* sequences as will be described here. These differences suggest that the *F.t. holarctica* subspecies may be more diverse than suggested to date, and also include polymorphisms which may contribute to the overall fitness of FTNF002-00 and clonal success of strains containing its genotype within its defined population niche.

## Results

### General Genome Features

An overview of the FTNF002-00 genome as well as the complete genomes of six other previously sequenced *F. tularensis* strains is presented in Table 1. FTNF002-00 possesses the smallest genome of the fully sequenced *F. tularensis* strains to date. Its genome is smaller by ∼5 MB as compared to the other two sequenced subsp. *holarctica* strains (Table 1). Incidentally, the same amount of difference (∼5.5 MB) exists between the genome sizes of the sequenced A.II strain and the two A.I strains (Table 1), while the sequenced strain of the *novicida* subspecies, which are non-pathogenic in humans, has a genome that is larger by about 11.5 MB than the largest genome among the pathogenic *F. tularensis* strains. The % G+C content of the different strains is approximately 32%, with slight variation between the subspecies. All seven strains listed in Table 1 encode identical numbers of tRNAs, rRNAs and other structural and regulatory RNAs. The numbers of pseudogenes as well as predicted proteins in the seven fully sequenced *F. tularensis* strains do not correlate with their genome size and vary considerably, likely due to the fact that many of these genomes have not been manually curated; it is noteworthy, however, that the larger non-virulent subsp. *novicida* genome contains substantially fewer predicted pseudogenes than the *F.t. tularensis* and *F.t. holarctica* genomes by comparison.

**Table 1:** Overview of general genome features of fully sequenced *F. tularensis* strains.^1^

	*Francisella tularensis*						
Subspecies	*holarctica*	*holarctica*	*holarctica*	*tularensis*	*tularensis*	*tularensis*	*novicida*
Genome Name	FTNF002-00	OSU18	LVS	FSC198	SCHU-S4	WY96-3418	U112
Accession #	NC_009749	NC_008369	NC_007880	NC_008245	NC_006570	NC_009257	NC_008601
Genome size^2^ (bp)	1,890,909	1,895,727	1,895,994	1,892,616	1,892,819	1,898,476	1,910,031
% G+C	32.161	32.158	32.152	32.26	32.259	32.268	32.475
% coding	87	74	82	79	78	79	89
Predicted proteins	1580	1555	1754	1605	1603	1634	1719
Pseudogenes	256	328	213	199	201	186	14
tRNAs	38	38	38	38	38	38	38
rRNAs^3^	10	10	10	10	10	10	10
Other RNAs^4^	5	5	5	5	5	5	5

1 Information presented here was obtained from the Genbank database.

2 Organisms are grouped by subspecies in order of ascending genome sizes. The first three are Type B strains; organisms 4, 5, and 6 are Type A strains. While 4 and 5 are subtype A.1 strains, organism 6 is subtype A.II strain.

3 In all seven organisms, the rRNA genes are organized in 3 operons of 16S, 23S, and 5S rRNAs and an additional orphan 5S rRNA. In *F. tularensis* subsp. *novicida* U112, one of the 5S rRNA that forms an operon with the 16S (FTN_1308) and the 23S (FTN_1305) rRNAs is not annotated and was found by BLAST search.

4 Each organism encodes a single molecule each of the RNA component of the ribonuclease P, a tmRNA, and a RFN element. Two copies of the RNA component of the signal recognistion particle (SRP) are encoded in each genome. Only 14 of the 35 strucural RNAs are annotated in the NCBI genbank database. Many of the missing structural RNAs could be identified in the genome using BLAST.

### Genomic organization

**Figure 1:**
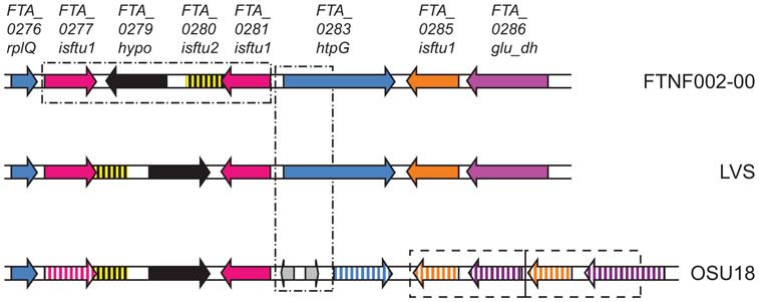
A 10 kb hypervariable genomic region carrying three regions of difference between the three *F. t. holarctica* genomes. Arrows indicate the direction of gene transcription. Genes and intergenic regions are not to scale. Homologous genes in the three genomes have been assigned the same color. Solid arrows indicate putative functional genes and hatched or grey arrows represent pseudogenes. The horizontal box on the FTNF002-00 chromosomal fragment indicates the 3,868 bp fragment (positioned at 247478–251345) that is inverted with respect to the other two genomes. A vertical box over the three genomes indicates the location of a 962 bp region that is different in OSU18 (see Table 2). The two equal sized horizontal boxes on the OSU18 genomic fragment indicate tandem duplications of a 1933 bp region (see Table 2).

The FTNF002-00 genome shares high sequence similarity (>99.9% on average) with the other two subspecies *holarctica* genomes, LVS and OSU18. The FTNF002-00 and the OSU18 genomes start at the same nucleotide bases, however the first nucleotide in the LVS genome maps to nucleotide position number 275 in both FTNF002-00 and OSU18. The organization and synteny of the FTNF002-00 genome is almost identical to that of the other subspecies *holarctica* genomes (data not shown) except for a single ∼3.8 kb inversion in the FTNF002-00 genome, with respect to both LVS and the original assembly of OSU18 (Fig. 1). Interestingly, OSU18 has subsequently been re-assembled and found to contain two unique inverted regions of ∼5 kb and ∼18 kb, respectively [26]. The single inversion in FTNF002-000 was probably caused by homologous recombination between two 874 bp identical inverted repeats that mark the two edges of the inverted genomic fragment. The inverted repeats overlap two ISFtu1 transposase ORFs (FTA_0277 and FTA_0281) at the two ends of the inverted fragment that encodes a single 331 aa long hypothetical protein (FTA_0279) that is as yet unique to *F. tularensis*. The protein bears weak sequence similarity to a short region within the pfam04488 glycosyltransferase sugar-binding region containing DXD motif. Multiple homologs of this protein occur in the sequenced genomes of the subsp. *tularensis* and *holarctica* strains. The FTNF002-00, LVS, and OSU18 genomes encode two, three, and three homologs, respectively. The genomes of two strains of subsp. *tularensis* subtype A.I, FSC198 and SCHU-S4, encode three homologs each, while the single completely sequenced genome of subtype A.II of the subsp. *tularensis* strain WY96-3418 encodes four copies of the hypothetical protein. It is interesting to note that the sequenced genome of subsp. *novicida* strain U112 does not contain a homolog of this hypothetical protein suggesting that the hypothetical protein may play a role in the pathogenic strains of *F. tularensis*.

### Regions of Difference (RD) within subspecies *holarctica*

**Figure 2:**
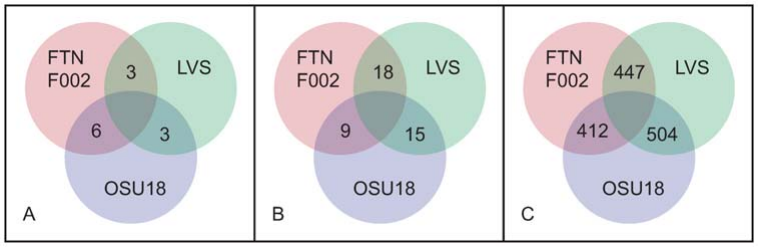
Overview of the shared and unique genomic differences in the three *F. t. holarctica* genomes. (A) Total numbers of regions of difference (RDs), (B) number of small insertions and deletions (indels), and (C) number of single nucleotide polymorphisms (SNPs) that are either shared or unique between the three strains. Further details of these differences are included in Tables 2, 3, and 4.

Each of the three *F.t. holarctica* genomes contain genomic regions that are absent in one or both of the other two subsp. *holartica* genomes (Fig. 2A). The FTNF002-00 genome revealed three regions that are different compared to LVS and OSU18 (Table 2). The first region, an approximately 1.5 kb deletion, termed the RD_23_ region, has been previously noted in FTNF002-00 [16]. The RD_23_ sequence encodes either hypothetical genes (in LVS) or pseudogenes (in OSU18), and may simply represent a non-functional region as it shows varying degrees of degradation in the different *F.t. holarctica* strains. However, it is worth noting that the RD_23_ deletion occurs in close proximity upstream of a regulator that is predicted to belong to the xenobiotic response element family of DNA-binding transcriptional regulators. Possibly, the RD_23_ deletion alters the expression of this transcriptional regulator in FTNF002-00.

**Table 2:** Large genomic regions of differences (RDs) between three *F. t. holarctica* genomes.^a^

#	Chromosomal location^b^	Details of the RD	Genes in the RD^c^
	A. RDs in FTNF002-00		
1	1034475̂1034476	RD23 (Dempsey et al., 2007).	FTH_1057 (hypothetical protein)
		A 1597 bp deletion in FTNF002-00	FTH_1058 (pseudogene)
			FTH_1059 (pseudogene)
2	1250392̂1250393	A 276 bp deletion in FTNF002-00	FTH_1284 (pseudogene)
3	1819665–1820026 (362 bp)	Replaces a 1626 bp region in LVS and OSU18.	FTH_1816 (conserved hypothetical)
	B. RDs in OSU18		
4	251346–252338 (993 bp)	Replaced by a sequence dissimilar 962 bp in OSU18.	FTA_0282 (pseudogene);
			FTA_0283 (htpG, heat shock protein 90)
5	253681̂253682	A tandem duplication of 1933 bp in OSU18.	FTH_0266 (pseudogene);
			FTH_0267 (pseudogene);
			FTH_0268 (glutamate dehydrogenase)
6	1844704–1844890 (187 bp)	Region deleted in OSU18.	FTA_2024 (ComEC/Rec2-related competence protein)
	C. RDs in LVS		
7	361986–362511 (526 bp)	Region deleted in LVS.	FTA_2144 (pseudogene);
			FTA_0415 (type IV pilus assembly protein)
8	407597–409077 (1481 bp)	Region deleted in LVS.	FTA_0461 (lipoprotein);
			FTA_0462 (hypothetical protein)
9	484148–484319 (172 bp)	Region deleted in LVS.	FTA_0522 (GMP kinases ATP-binding protein)
10	1041146–1041356 (211 bp)	Region deleted in LVS.	FTH_1057 (conserved hypothetical);
			FTH_1058 (pseudogene);
			FTH_1059 (pseudogene)
11	1169860̂1169861	A tandem duplication of 468 bp in LVS.	FTL_1219 (hypothetical)
12	1392027̂1392028	A tandem duplication of 3914 bp in LVS.	FTL_1467 (ISFtu2);
			FTL_1468 (ATP-dependent metalloprotease);
			FTL_1469 (pseudogene);
			FTL_1470 (pseudogene);
			FTL_1471 (ISFtu2)

a Regions of differences larger than 100 bp in the genomes of FTNF002-00, LVS, and OSU18 are listed.

b Coordinates of the RDs correspond to the chromosomal positions in the FTNF002-00 genome. Length of the RD is provided in parenthesis if the sequence occurs in FTN002-00, while a “ ^” indicates the position of a sequence that is absent in the FTN002-00 genome.

c Locus tags of genes contained in or overlapping the RD are listed; FTA  = > FTNF002-00, FTL  = > LVS, and FTH  = > OSU18 genomes.

A second small genomic deletion of 276 bp occurs within a pseudogene (FTA_1387) in FTNF002-00. The corresponding genes in OSU18 (FTH_1284) as well as in all three sequenced subspecies *tularensis* genomes are also pseudogenes, suggesting that the protein is dispensable for pathogenicity. The third region reveals that a 362 bp fragment, at 1819665–1820026 in the FTNF002-00 chromosome, replaces a second genomic region of about 1.6 kb found in LVS and OSU18. The two edges of the fragment partially overlap transposase sequences on either side. This fragment along with the flanking transposase genes (a total of 2.9 kb region) occurs in 3 copies in both LVS and OSU18, and only occurs in two copies in FTNF002-00. Interestingly, one of the copies in FTNF002-00 occurs in the inverted genomic region (see above), suggesting that the genomic inversion and the deletion events may have been linked evolutionarily.

Next, FTNF002-00 and LVS share three genomic regions that are different in OSU18 (Fig. 2A, Table 2), one of which involves a ∼2 kb tandem duplication in OSU18. Of the remaining two, one region (993 bp) is immediately downstream of the inverted fragment in FTNF002-00 (see Fig. 1) and is predicted to encode a small hypothetical protein (FTA_0282) and overlaps a 628 aa chaperone protein, HtpG (FTA_0283). This ∼1 kb sequence occurs in single copy in FTNF002-00, LVS, all three subsp. *tularensis* genomes as well as in subsp. *novicida* U112. However, the corresponding region in OSU18 is replaced by a completely different sequence 962 bp long that carries partial sequence of ISFtu1 and encodes two divergently-oriented pseudogenes (Fig. 1). The other, a 187 bp fragment shared by FTNF002-00 and LVS, but deleted in OSU18, occurs in the middle of an ORF predicted to encode a 671 aa competence-related protein. Homologs of this protein are predicted in LVS, subsp. *tularensis* A.II strain WY96-3418, as well as in subsp. *novicida* strain U112; however, genomes of the two subsp. *tularensis* A.I strains (FSC198 and SCHU-S4) contain pseudogenes carrying a frameshift mutation.

Further, there are a total of six genomic fragments in FTNF002-00 that are shared with OSU18, but absent in LVS (Table 2), two of which include tandemly duplicated regions in LVS. The remaining four include: (a) an approximately 1.5 kb region (at 407597–409077); (b) a ∼0.5 kb fragment (at 361986–362511); (c) a ∼0.2 kb fragment (at 484148–484319); and (d) a ∼0.2 kb fragment (at 1041146–1041356). Fragment (c) above is also lacking in the subtype A.I subsp. *tularensis* genomes.

Lastly, as mentioned above, both LVS and OSU18 each possess tandem duplications in certain regions that are found only in one copy in the other two genomes (Table 2). The first region involves a 1933 bp fragment in OSU18, carrying a copy of *ISFtu1*, that is not present in the corresponding regions in the LVS and FTNF002-00 genomes. This region occurs in two tandem copies in OSU18 and only the second copy is shared in OSU18, LVS and FTNF002-00. Two other regions occur as tandem duplications in LVS but in single copy in the other *F. tularensis* genomes. One of these regions in LVS involves tandem duplication of a ∼3.9 kb long fragment (at 1392385–1396298 in the LVS chromosome). Only one copy of the 3.9 kb region occurs in the other six sequenced *F. tularensis* genomes. The second region involves tandem duplication of a 468 bp fragment in LVS (at 1169486–1169953 in the LVS chromosome) and overlaps the 5′ end of a gene encoding a hypothetical protein.

Despite these regions of difference, there are no genomic regions where all three subsp. *holarctica* strains are different.

### Small Insertions and Deletions

In order to identify the unique variations in FTNF002-00 as compared to those of LVS and OSU18, we performed a detailed analysis of small stretches of insertions and deletions. Forty-two short sequences ranging from 10–100 nucleotides were identified that were either inserted or deleted in one or more of the three genomes compared (Fig. 2B, Table 3). Of these, fifteen regions are different in FTNF002-00, while 18 are different in OSU18, and 9 in LVS. Eleven of the fifteen short sequences are absent in the corresponding position in the FTNF002-00 genome, while four short sequences are lacking in the other two *holarctica* genomes. The insertions/deletions are either mostly in intergenic regions (five) or in genes that are either pseudogenes (two) or ORFs encoding hypothetical proteins (two) in the *holarctica* genomes. However, interestingly, two deletions appear to inactivate two distinct genes in FTNF002-00 causing them to become pseudogenes specifically in FTNF002-00. The first one is a frame-shifting deletion of 16 nucleotides within the FTA_1097 pseudogene, the corresponding functional gene in OSU18 encodes a 188 amino acid long protein (FTH_1016) of unknown function that is conserved in all the fully sequenced subsp. *holarctica* as well as subsp. *tularensis* strains. The second deletion of 20 bp also results in a frame-shifted pseudogene (FTA_1713) whose functional counterpart in OSU18 (FTH_1570) encodes a transporter of the major facilitator superfamily (MFS). This transporter is predicted to be a drug:H^+^ antiporter involved in the efflux of drugs and antimicrobial compounds.

**Table 3:** Small insertions and deletions (Indels) in the *holarctica* genomes.^a^

	Chromosomal location^b^	Details of the region	Genes/Gene products affected by the insertion/deletion.^c^
	A. Indels in FTNF002-00		
1	139735̂139736	17 bp deletion in FTNF002-00	Hypothetical proteins - FTA_0145, FTL_0134, FTH_0125.
2	141071̂141072	16 bp deletion in FTNF002-00	None; intergenic sites in all three genomes
3	205171̂205172	54 bp deletion in FTNF002-00	TPR repeat-containing protein - FTA_0221, FTL_0205, FTH_0200.
4	307294–307366 (73 bp)	Insertion in FTNF002-00	Hypothetical protein - FTA_0340. Pseudogenes - FTL_0321, FTH_0321.
5	409906̂409907	16 bp deletion in FTNF002-00	None; intergenic sites in all three genomes
6	725732̂725733	48 bp deletion in FTNF002-00	Pseudogene - FTA_0775, FTL_0734, FTH_0737.
7	733922̂733923	70 bp deletion in FTNF002-00	Pseudogene - FTA_0784, FTH_0744. LysR family regulator - FTL_0742.
8	914224̂914225	16 bp deletion in FTNF002-00	None; intergenic sites in all three genomes
9	996737̂996738	16 bp deletion in FTNF002-00	Pseudogene - FTA_1097. Hypothetical proteins - FTL_1037, FTH_1016.
10	1168125̂1168126	27 bp deletion in FTNF002-00	None; intergenic sites in all three genomes
11	1512625–1512646 (22 bp)	Insertion in FTNF002-00	Pseudogene - FTA_1673, FTH_1534. Mechanossensitive ion channel - FTL_1588.
12	1550402̂1550403	20 bp deletion in FTNF002-00	Intergenic site - FTNF002-00. MFS transporters - FTL_1624, FTH_1570.
13	1818821–1818836 (16 bp)	Insertion in FTNF002-00	Intergenic sites - FTNF002 & OSU18. Pseudogene - FTL_1890.
14	1867991–1868006 (16 bp)	Insertion in FTNF002-00	None; intergenic sites in all three genomes
15	1875603̂1875604	25 bp deletion in FTNF002-00	Pseudogenes - FTA_2064, FTL_1953, FTH_1870.
	B. Indels in OSU18		
16	15760–15786 (27 bp)	Deletion in OSU18	Pseudogenes - FTA_0021, FTL_0017, FTH_0017.
17	63536–63580 (45 bp)	Deletion in OSU18	Pseudogenes - FTA_0073, FTL_0064. No gene in OSU18.
18	175448̂175449	25 bp insertion in OSU18	UDP-N-acetylmuramate-alanine ligase - FTA_0188, FTL_0172. Pseudogene - FTH_0166.
19	264253–264282 (30 bp)	Deletion in OSU18	CDP-alcohol phosphatidyltransferase - FTA_0295, FTL_0278, FTH_0277.
20	491032–491059 (28 bp)	Deletion in OSU18	Transposase ISFTu1 - FTA_0529, FTL_0503. Pseudogene - FTH_0500.
21	597826–597853 (28 bp)	Deletion in OSU18	Transposase ISFTu1 - FTA_0641, FTL_0607. Pseudogene - FTH_0608.
22	621670–621697 (28 bp)	Deletion in OSU18	Transposase ISFTu1 - FTA_0664, FTL_0631. Pseudogene - FTH_0634.
23	655230̂655231	21 bp insertion in OSU18	ATP-binding trancriptional regulator - FTA_0703, FTL_0668, FTH_0671.
24	667720–667747 (28 bp)	Deletion in OSU18	Transposase ISFTu1 - FTA_0712, FTL_0678. Pseudogene - FTH_0680.
25	671929–671956 (28 bp)	Deletion in OSU18	Transposase ISFTu1 - FTA_0719, FTL_0682. Pseudogene - FTH_0684.
26	937312̂937313	26 bp insertion in OSU18	None; intergenic sites in all three genomes
27	1124775–1124785 (11 bp)	Deletion in OSU18	None; intergenic sites in all three genomes
28	1286947–1287001 (55 bp)	Deletion in OSU18	Hypothetical protein - FTA_1431, FTL_1353. Pseudogene - FTH_1316.
29	1295739–1295754 (16 bp)	Deletion in OSU18	None; intergenic sites in all three genomes
30	1489823–1489842 (20 bp)	Deletion in OSU18	None; intergenic sites in all three genomes
31	1498373–1498445 (73 bp)	Deletion in OSU18	Pseudogene - FTA_1660, FTH_1522. Hypothetical protein - FTL_1575.
32	1695061–1695074 (14 bp)	Deletion in OSU18	Cytochrome ubiquinol oxidase - FTA_1870. Pseudogene - FTL_1764, FTH_1703.
33	1875118̂1875119	16 bp insertion in OSU18	None; intergenic sites in all three genomes
	C. Indels in LVS		
34	424816̂424817	19 bp insertion in LVS	None; intergenic sites in all three genomes
35	799919–799929 (11 bp)	Deletion in LVS	Pseudogene - FTA_0855, FTH_0803. No gene predicted in LVS.
36	1069708–1069766 (59 bp)	Deletion in LVS	Hypothetical protein - FTA_1184, FTL_1123, FTH_1097.
37	1274235-1274260 (26 bp)	Deletion in LVS	Pseudogene - FTA_1416, FTH_1304. Oligopeptide transporter -FTL_1339.
38	1445437̂1445438	12 bp insertion in LVS	Chitinase - FTA_1605, FTL_1521, FTH_1471.
39	1492926–1492944 (19 bp)	Deletion in LVS	None; intergenic sites in all three genomes
40	1691485–1691524 (40 bp)	Deletion in LVS	Pseudogene - FTA_1866, FTH_1700. No gene predicted in LVS.
41	1702165–1702257 (93 bp)	Deletion in LVS	Hypothetical protein - FTA_1879, FTL_1773, FTH_1709.
42	1827681–1827692 (12 bp)	Deletion in LVS;	Pseudogene - FTA_2008, FTH_1823. Hypothetical protein - FTL_1901.
		Only 6 bp left in OSU18.	

a Insertions and deletions smaller than 100 bp and larger than 10 bp in the genomes of FTNF002-00, LVS, and OSU18 are listed.

b The fragments are listed in an ascending order of their location in FTNF002-00 genome. Length of the fragment is provided in parenthesis if the sequence occurs in FTN002-00, while a “ ^” indicates the position of a sequence that is absent in the FTN002-00 genome.

c Locus tags of genes contained in or overlapping the fragment are listed; FTA  = > FTNF002-00, FTL  = > LVS, and FTH  = > OSU18 genomes.

The OSU18 genome contains fourteen small deletions and four small insertions compared to the FTNF002-00 and LVS genomes (Table 3). Almost half of the insertions/deletions are either in intergenic regions or in pseudogenes. Interesting in-frame insertions and deletions are also observed in two OSU18 proteins. One is a 30 bp deletion in the *pgsA* gene that is predicted to encode a phosphatidylglycerophosphate synthase (FTH_0277), which is shorter by 10 amino acids than the homologs in LVS and FTNF002-00. The PgsA protein in other organisms is known to be important in the biogenesis of membrane lipids. The second sequence, an in-frame insertion of 21 bp in the ATP-dependent DNA helicase gene (FTH_0671), adds a copy of a 7 aa repeat sequence, ESSPKEQ, in the 483 aa long protein in OSU18. It is worthwhile to note that homologs of this protein in the other pathogenic strains of *Francisella* are shorter (476 aa), while subsp. *novicida* GA99-3548 encodes a longer (490 aa) protein. In addition, two more sequence alterations in OSU18 inactivate two separate genes. A 25 bp insertion in the UDP-N-acetylmuramate-L-alanine ligase gene (FTH_0166) of OSU18 appears to cause frameshift mutations that are predicted to inactivate the gene. The ligase is known to be important in the biosynthesis of peptidoglycan in the bacterial cell wall in other bacteria. Another 55 bp deletion causes a pseudogene (FTH_1316) that is supposed to encode a 97 aa protein of unknown function. This small protein is encoded in FTNF002-00 (FTA_1431) and is conserved in other *Francisella* genomes. The gene encoding this hypothetical protein is only 23 nucleotides downstream of the gene that encodes the DNA replication and repair protein RecF protein (FTA_1430) in FTNF002-00 as well as in LVS. The short intergenic distance suggests that the two genes are part of a single operon and that they are involved in a common metabolic function - DNA replication and repair.

### Single Nucleotide Polymorphisms

Comparative alignments between regions shared between all three *holarctica* genomes revealed 1364 polymorphic sites. Surprisingly, only a single site was found where each of three genomes contained a different nucleotide. This site occurs at position 731044 in FTNF002-00 and forms the third base of the codon encoding Alanine-175 in the glucose-inhibited division protein A (FTA_0780). The FTNF002-00, LVS, and OSU18 genomes contain T, G, and A, respectively, at the corresponding positions. The mutation causes no change in amino acid. Of the remaining 1363 polymorphic sites, six occur within the regions of difference discussed above, 504 sites contained a different nucleotide in FTNF002-00 as compared to LVS and OSU18, while LVS alone carried a different nucleotide at 412 sites, and OSU18 alone showed differences at 447 sites (Fig. 2C).

Among the 504 polymorphic sites in FTNF002-00, 172 sites are either intergenic (85 sites) or are within a gene (87 sites) but cause no change in amino acid in the encoded protein. However, 103 polymorphic sites in FTNF002-00 cause amino acid mutations in 99 proteins (Table 4). Approximately 20% of these mutations change the amino acid to an isoleucine in FTNF002-00 and the most frequent change is from valine in LVS and OSU18 to isoleucine in FTNF002-00. Based on the BLOSUM62 matrix used for scoring BLAST search alignments, the V→I change is a conserved amino acid change. Using the BLOSUM62 matrix, the most non-conservative amino acid replacements in FTNF002-00 were identified to be R→C, P→L, R→I, G→C, I→N, and D→Y mutations. Interestingly, the above non-conserved amino acid changes predominantly occurred in proteins that function in the essential cellular functions such as replication, transcription, translation, ATP and cell wall synthesis. Notably, using this approach, the intracellular growth locus, subunit D protein was also identified as one of the proteins carrying a non-conserved C→F replacement in FTNF002-00 compared to the other two subsp. *holarctica* genomes.

**Table 4:** List of 99 FTNF002-00 proteins with amino acid mutations compared to LVS and OSU18.

Locus tag	AA change	Score	Predicted Protein Product	COG
FTA_1502	D→Y	−3	hypothetical protein	
FTA_0649	G→C	−3	DNA-directed RNA polymerase subunit alpha	K
FTA_1324	I→N	−3	DEAD/DEAH box helicase family protein	L
FTA_0068	P→L	−3	hypothetical protein	S
FTA_0250	P→L	−3	elongation factor G	J
FTA_0810	P→L	−3	hypothetical protein	Q
FTA_1019	R→C	−3	tyrosyl-tRNA synthetase	J
FTA_1197	R→C	−3	hypothetical protein	G
FTA_1908	R→C	−3	F0F1 ATP synthase subunit A	C
FTA_1916	R→C	−3	translation initiation factor IF-2	J
FTA_2070	R→C	−3	aminopeptidase N	E
FTA_1085	R→I	−3	radical SAM enzyme, Cfr family protein	R
FTA_1825	R→I	−3	glutamyl-tRNA reductase	H
FTA_0122	C→F	−2	intracellular growth locus subunit D	
FTA_1223	C→F	−2	intracellular growth locus DII	
FTA_2061	C→Y	−2	hypothetical protein	R
FTA_1125	G→E	−2	fumarylacetoacetate hydrolase family protein	Q
FTA_0793	R→L	−2	hypothetical protein	S
FTA_0589	S→F	−2	ribonuclease R	K
FTA_1078	S→F	−2	coproporphyrinogen III oxidase	H
FTA_0244	S→I	−2	undecaprenyl pyrophosphate synthetase	I
FTA_0840	S→L	−2	adenylate kinase	F
FTA_1584	S→L	−2	sulfate permease family protein	P
FTA_0210	Y→C	−2	protoheme IX farnesyltransferase	O
FTA_0123	Y→N	−2	hypothetical protein	
FTA_0097	D→G	−1	acetyltransferase protein	I
FTA_0919	D→G	−1	hypothetical protein	
FTA_0329	G→D	−1	dihydrolipoamide acetyltransferase	C
FTA_1126	G→D	−1	phosphorylase family 2 protein	F
FTA_0510	I→T	−1	phosphoglucomutase	G
FTA_1126	M→R	−1	phosphorylase family 2 protein	F
FTA_1286	P→S	−1	hypothetical protein	K
FTA_0658	P→T	−1	glycosyl transferase	M
FTA_1509	S→R	−1	HAD family hydrolase	R
FTA_1892	T→I	−1	succinate dehydrogenase, flavoprotein subunit	C
FTA_1560	V→F	−1	prepilin-type N-terminal cleavage/methylation domain-containing protein	
FTA_0591	A→T	0	short chain dehydrogenase/reductase family oxidoreductase	I
FTA_0724	A→T	0	NAD+ synthetase	H
FTA_0931	A→T	0	hypothetical protein	
FTA_1057	A→T	0	DNA polymerase III, epsilon subunit	L
FTA_1101	A→T	0	polyprenyl synthetase family protein	H
FTA_1911	A→T	0	ribosomal large subunit pseudouridine synthase C	J
FTA_1966	A→T	0	phosphoribosylformylglycinamidine synthase	F
FTA_0069	A→V	0	hypothetical protein	R
FTA_0187	A→V	0	tetrapyrrole (corrin/porphyrin) methylase family protein	R
FTA_0648	A→V	0	recombination factor protein RarA	L
FTA_0746	A→V	0	hypothetical protein	
FTA_0809	A→V	0	gamma-glutamyltransferase	E
FTA_0877	A→V	0	type II secretion system protein/type IV-A pilus assembly ATPase PilB	N
FTA_0916	A→V	0	sugar isomerase domain-containing protein	M
FTA_1339	A→V	0	alpha/beta hydrolase fold protein	I
FTA_1435	A→V	0	UTP–glucose-1-phosphate uridylyltransferase	M
FTA_0702	F→L	0	hypothetical protein	S
FTA_1844	F→L	0	O-sialoglycoprotein endopeptidase	O
FTA_0102	G→S	0	putative chitinase	G
FTA_0594	G→S	0	cyclopropane-fatty-acid-phospholipid synthase	M
FTA_0901	G→S	0	preprotein translocase subunit SecF	U
FTA_0793	K→N	0	hypothetical protein	S
FTA_1344	K→N	0	adenosylmethionine-8-amino-7-oxononanoate aminotransferase	H
FTA_1828	L→F	0	drug resistance transporter, Bcr/CflA family protein	G
FTA_1589	T→A	0	transposase ISFtu1	L
FTA_0467	V→A	0	hypothetical protein	S
FTA_0469	A→S	1	FMN reductase, NADPH-dependent	R
FTA_1106	A→S	1	D-alanyl-D-alanine carboxypeptidase/D-alanyl-D-alanine-endopeptidase	M
FTA_1611	A→S	1	phosphopyruvate hydratase	G
FTA_0592	D→N	1	hypothetical protein	
FTA_0900	D→N	1	protein-export membrane protein SecD	U
FTA_1127	D→N	1	tRNA pseudouridine synthase A	J
FTA_1186	D→N	1	HipA domain-containing protein	R
FTA_1566	D→N	1	putative permease YjgP/YjgQ family protein	R
FTA_0149	E→K	1	hypothetical protein	
FTA_0217	E→K	1	hypothetical protein	R
FTA_0317	E→K	1	N-acetylmuramic acid-6-phosphate etherase	R
FTA_0377	E→K	1	chorismate pyruvate lyase	H
FTA_1521	E→K	1	sugar isomerase family protein	R
FTA_2061	E→K	1	hypothetical protein	R
FTA_0587	H→N	1	ribonuclease III	K
FTA_0126	I→M	1	hypothetical protein	
FTA_0487	M→I	1	hypothetical protein	
FTA_1086	M→I	1	TPR repeat-containing protein	N
FTA_1571	M→I	1	tRNA-(ms(2)io(6)a)-hydroxylase	F
FTA_1828	M→I	1	drug resistance transporter, Bcr/CflA family protein	G
FTA_0842	N→S	1	Type IV pili associated protein	
FTA_1848	N→S	1	DNA-directed RNA polymerase subunit beta	K
FTA_0880	Q→K	1	UDP-N-acetylmuramyl tripeptide synthase	M
FTA_0853	V→L	1	major facilitator transporter	G
FTA_1688	V→L	1	delta-aminolevulinic acid dehydratase	H
FTA_0052	V→M	1	transglutaminase-like superfamily protein	E
FTA_1460	K→R	2	putative carbohydrate kinase/YjeF-like protein	S
FTA_1029	L→I	2	putative N-6 DNA methylase	V
FTA_0143	R→K	2	pyruvate phosphate dikinase	G
FTA_0505	R→K	2	glycine dehydrogenase subunit 1	E
FTA_1565	Y→H	2	putative permease, YjgP/YjgQ family protein	R
FTA_0096	V→I	3	acetyltransferase protein	I
FTA_0422	V→I	3	phosphoribosylaminoimidazole carboxylase ATPase subunit	F
FTA_0429	V→I	3	2-polyprenylphenol 6-hydroxylase	R
FTA_0537	V→I	3	transposase ISFTu1	L
FTA_0711	V→I	3	DNA ligase	L
FTA_1237	V→I	3	hypothetical protein	O
FTA_1312	V→I	3	phospho-2-dehydro-3-heoxyheptonate aldolase	E
FTA_1313	V→I	3	transposase ISFtu1	L
FTA_1626	V→I	3	AMP-binding domain-containing protein	I
FTA_1922	V→I	3	transposase ISFtu1	L

103 amino acid mutations affect 99 proteins in FTNF002-00. The four proteins that contain two mutations each are FTA_0793, FTA_1126, FTA_1828, and FTA_2061.

The list is sorted in the ascending order of the score in column 3. The score for amino acid change is based on the BLOSUM62 scoring matrix. A lower score indicates a non-conserved change, while a higher score indicates a conserved change.

The amino acid change in column 2 represents a change from an amino acid that occurs in LVS and OSU18 protein to the amino acid in the FTNF002-00 ortholog.

The COG category in the last column represents the single letter code for the functional COG categories (http://www.ncbi.nlm.nih.gov/COG/grace/fiew.cgi).

## Discussion

As previously noted, the FTNF002-00 genome shares >99.9% sequence similarity with the LVS and OSU18, and the organization of the three genomes appears to be largely similar with a few exceptions. FTNF002-00 is the first fully sequenced genome of *F.t. holarctica* carrying RD_23_. The strain was isolated from a French patient with *F.t. holarctica* bacteremia. Strains carrying RD_23_ are believed to be highly clonal and recently emerged in France and the Iberian Peninsula, and are virulent like FTNF002-00 as demonstrated by their association with the numerous human cases from the 1997–1998 Spanish outbreak. With respect to relative virulence of the subsp. *holarctica* strains compared in this analysis, LVS is attenuated, whereas both OSU18 and FTNF002-00 represent virulent strains, the former isolated from a dead beaver, and the latter from a human with *F.t. holarctica* bacteremia as mentioned above. Due to the uniqueness of FTNF002-00 based on the presence of the RD_23_ deletion and its observed pathogenic success in its geographic niche, it is important to thoroughly examine its genomic differences with respect to other sequenced *F.t. holarctica* genomes to understand the genomic traits that define this pathogen. Our analyses showed that the FPI-associated *pdpD* gene in FTNF002-00 is truncated and present as a pseudogene as it is in the other *F.t. holarctica* genomes, and no obvious differences in known *F. tularensis* virulence genes were found in the FTNF002-00 genome (data not shown). However, we did identify interesting features that include several potentially new loci that may possibly contribute to its geographically defined fitness and pathogenic success.

The smaller genome size of FTNF002-00 is interesting. Genome reduction is a hallmark of intracellular pathogens. Presumably, progressive reduction or reorganization of the genome, such that can inactivate non-essential genes resulting in net increase in the number of pseudogenes, provides optimal genotype for enhanced replication and growth of the pathogen, evasion of host immune response, and increased expression of the pathogen's virulence arsenal. This has been shown by comparative genome analyses among strains within the *Mycobacterium tuberculosis* complex and *Yersinia* species [27], [28]. For example, the more virulent etiologic agent of plague, *Y. pestis*, is believed to have evolved from the less virulent enteric pathogen, *Y. pseudotuberculosis*; this process appears to have involved a massive net inactivation/loss of genes and substantial gain of pseudogenes as shown within the *Y. pestis* genome as compared with *Y. pseudotuberculosis* [28]. Although, experimental evidences lack behind to successfully elucidate the relationship between genomic deletions/reorganization and the virulence potential of *F. tularensis* strains, the observed difference in the genome size of the non-pathogenic subsp. *novicida* and the pathogenic *F.t. tularensis* and *F.t. holarctica* strains is intriguing. Furthermore, the smaller genome of the more potent A.I pathogen than the A.II type [8] is suggestive of a linkage between the two properties. In this regard, when compared to the other two subsp. *holarctica* genomes, the RD_23_ deletion as well as the overall size reduction of FTNF002-00 as accounted for by the combination of RDs and insertions/deletions (Tables 2–3) observed within its genome may be a result of natural selection towards enhancing its survival, replication, and disease potential; but this would require experimental evaluation.

The RD_23_ deletion in FTNF002-00 may alter expression of the xenobiotic response element family transcriptional regulator, given the proximity of the two. While the function of this family of regulators remains poorly understood in bacteria, studies suggest their involvement in stress response. Enhanced response to stress could conceivably be a selective trait, especially in intracellular pathogens. Reversed orientation of one of the two noted pfam04488 homologs in FTNF002-00 caused by the 3.8 kb inversion may also play a role in its virulence as compared to other *F.t. holarctica* strains, perhaps due to altered expression or regulation of that gene or surrounding region. For example, downstream of the inverted region is the 1 kb RD in common with LVS, but not OSU18, predicted to encode the 628 aa HtpG chaperone protein, which itself may play a role in acclimation or fitness of FTNF002-00, even though it is likely inconsequential in LVS due the strain's attenuated state. HtpG has been shown to have a role in virulence in *Porphyromonas gingivalis* [29], and more recently has been demonstrated to be essential for survival and growth *in vivo* by *F. t.* subsp. *novicida* [30].

Further, the predicted longer *pgsA* gene in FTNF002-00 as compared with its 10 aa shorter homolog in OSU18 may have an important role in its pathogenic potential, especially considering the protein's role in other organisms involving biogenesis of membrane lipids, which may alter host-pathogen interactions. Likewise intriguing are potential differences in the function of some of the other genes, to include the hypothetical protein associated with the operon encoding the RecF protein and subsequent involvement in DNA replication and repair, as well as the 99 proteins in FTNF002-00 affected by amino acid changes due to SNPs. Many of these polymorphisms, especially the ones noted in genes involved in virulence and essential cellular functions, may contribute to FTNF002-00's overall fitness and pathogenic competence.

While our analyses of FTNF002-00 show numerous polymorphisms with respect to the other *F.t. holarctica* sequences compared and support the hypothesis that the subspecies is genetically more diverse than previously described based on a whole-genomic scale, another point of interest involves the evolutionary relationship between FTNF002-00, OSU18, and LVS. Vogler *et al*. recently performed detailed SNP analysis and multi-locus variable numbered tandem repeat analysis (MLVA) of a set of 496 globally diverse *F. tularensis* isolates [17]. Their findings correlate with the marked diversity observed from our comparative CGS analysis approach, and together these data provide possible clues about the evolutionary relationship. For example, the hypothesis that *F.t. holarctica* originated in N. America and subsequently spread to Eurasia is supported based on the presence of both N. American and Scandinavian strains within the subclade defined by SNPs derived from the OSU18 CGS [17]. The analysis tends to suggest that subclades defined by SNPs from FTNF002-00 and LVS diverged from subclades of Scandinavian origin, and corroborates previous findings [16] indicating that the FTNF002-00 subclade emerged very recently.

**Figure 3:**
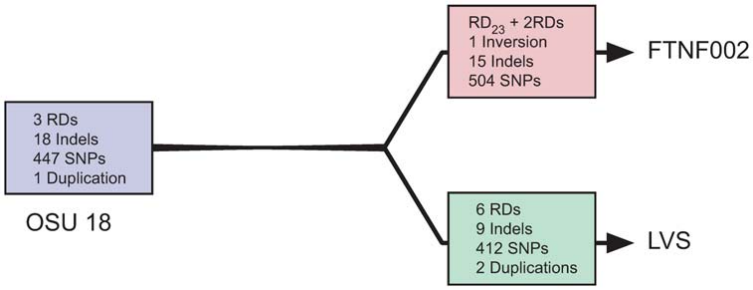
A genome-based model depicting the divergence of the FTNF002 and LVS from the more ancestral OSU18.

Using our comparative CGS analysis method, we can hypothesize the evolutionary events leading to the divergence of the FTNF002-00, OSU18, and LVS strains by comparing the polymorphisms between the genomes (Fig. 3). Our CGS analyses reveal likely genomic events that may have led to the emergence of the LVS and FTNF002 subclades. In the three genomes compared, the number of genomic changes observed in each of the three genomes is substantially the same (Fig. 2). This leads us to propose a model for the divergence of LVS and FTNF002 from the more ancestral OSU18 (Fig. 3). As is evident, large and small deletions, insertions, duplications, as well as SNPs have shaped the emergence of the two subclades. Availability of more genomes within each subclade in the future may provide more resolution to this evolutionary model.

Whereas SNP and analysis by other methods such as MLVA provide very robust phylogeographic genetic differentiation, comparative CGS analysis is essential to provide detailed genetic comparisons at the level of potential gene expression, such that may define pathogenicity or fitness, as presented by our analyses. Vogler *et al.* observed the presence of several MLVA genotypes for most of the SNP-defined subclades [17], and this suggests that there may be substantial genetic diversity at the CGS scale. Such potential genomic diversity may be essential to understanding the fitness of relative genotypes within the subclades, and this should be further evaluated by sequencing and comparing more representative strains. If a consensus of genetic features thus far unique to FTNF002-00 can be established among additional strains within the subclade, then it may serve as a basis for studying the functional aspects of those features as pertains to clonal success including pathogenicity of the FTNF002-00 subclade.

## Materials and Methods

### Strain Cultivation and Nucleic Acid extraction

*F. tularensis* strain FTNF002-00 was propagated on chocolate agar at 37°C in 5% CO_2_. Glycerol fermentation characteristics were determined using Biolog® (Biolog, Inc., Hayward, CA). In addition to biochemical characteristics, FTNF002-00 was tested for subspecies-specific genome alterations by PCR using previously published primers specific for RD*_tularensis_*1 [9]. High quality DNA was extracted using a large-scale bacterial genomic DNA preparation method [31] with omission of the CsCl step. These operations strictly complied with Biosafety in Microbiological and Biomedical Laboratories (BMBL), 4th Edition [32], which also encompasses 42-Code of Federal Regulations (CFR) Part 73 to govern the possession, use, and transfer of select agents. Site-specific standard operating procedures (SOPs) regulated such operations to include entry and exit, waste disposal, personal protective equipment (PPE) gowning and disrobing, occupational-health surveillance, and post-extraction DNA non-viablility certification.

### Genome Sequencing

The random shotgun method was used in sequencing the genome of *F. tularensis* FTNF002-00. Medium (8 kb) and small (3 kb) insert random sequencing libraries in pMCL200 and pUC-hinc cloning vectors (respectively) were subjected to paired-end Sanger sequencing to an average depth of 19× coverage. After the shotgun stage, reads were assembled with parallel phrap (High Performance Software, LLC). Possible mis-assemblies were corrected with Dupfinisher (unpublished, C. Han) or transposon bomb of bridging clones (EZ-Tn5 <P6Kyori/KAN-2> Tnp Transposome kit, Epicentre Biotechnologies). Gaps between contigs were closed by editing, custom primer walks or PCR amplification. The completed genome sequence of *F. tularensis* FTNF002-00 was finished to a standard of less than 1 error in 100,000 bases.

### Gene prediction and Annotation

Genome annotation was performed at BioHealthBase (www.biohealthbase.org) using TIGR's gene prediction algorithm, Glimmer3 [33], automated annotation pipeline, AutoAnnotate [34], followed by manual curation using Manatee [34]. The genome sequence was reformatted so that dnaA was the first gene and sequence coordinates were reassigned before submitting the sequence for gene predictions. Glimmer 3.02 was used to predict protein coding genes using default parameters. The genes coding for tRNAs were identified using tRNAscanSE [35] (version 1.23, parameters: -B). A sequence similarity search using Exonerate [36] (version 1.0.0, parameters: -m NER) was used to identify 16S and 23S ribosomal RNA genes. A sequence similarity search against RFam [37] (release 7.0, rfam_scan.pl version 0.1, parameters: –bt 0.1 –nobig), a comprehensive database of non-coding RNA (ncRNA) families was used to identify genes coding for other non-coding RNAs, (such as 5S ribosomal RNAs).

For assignment of protein function, the first step was a sequence similarity search BLAST [38] for each of the predicted protein against a non-redundant amino acid database (nraa) made up of all proteins available from GenBank, PIR and SWISS-PROT using the BLAST-Extend-Repraze (BER) algorithm. All of the proteins from the genome sequence were also searched against the Pfam [39] (release 20.0) HMMs (hmmpfam HMMER version 2.3.2, parameters: -E 0.1 –cut_ga) and TIGRFAMs [40] (release 6.0) built from highly curated multiple alignments of proteins thought to share the same function or to be members of the same family. Each HMM has an associated cutoff score above which hits are known to be significant. Additional data used to support annotations included prediction of trans-membrane helices using TMHMM [41], prediction of signal peptide with signalP [42], lipoprotein motif and Clusters of Orthologous Groups (COG) [43]. These data used by AutoAnnotate to make functional predictions for proteins were then made available in the Manatee interface for manual evaluation of the predicted function. Manual curation involved correcting the start sites for the predicted ORFs, reviewing overlapping ORFs, frameshifts and premature stops, analysis of predicted pseudogenes, transposons and transport proteins.

The complete genome sequence of FTNF002-00 has been deposited in Genbank under the accession number CP000803 and the Refseq accession is NC_009749.

### Data acquisition and Analysis

Genome sequence files of completely sequenced genomes of seven *Fransicella tularensis* strains listed in Table 1 were obtained from the NCBI ftp site (ftp://ftp.ncbi.nih.gov/genomes/Bacteria/) in September 2007. The executable BLAST (Altschul et al., 1997) programs were obtained from the National Center for Biotechnology Information (NCBI) (http://www.ncbi.nlm.nih.gov/BLAST/download.shtml). The complete genome and predicted proteome of FTNF002-00 were analyzed against those of the other two *F. tularensis* subsp. *holarctica* genomes, LVS and OSU18. Whole genome and whole proteome analyses were performed locally using BLAST programs. Results of whole genome DNA BLAST search (filtering was set to off) were used to carefully align the whole genomes of FTNF002-00 with either LVS or OSU18 genomes. Genomic regions of differences and polymorphic sites were identified from the aligned genomes using short perl codes.
